# The role of pancreatic islet macrophages in type 2 diabetes mellitus: from underlying pathological mechanisms to therapeutic target discovery

**DOI:** 10.1016/j.metop.2025.100418

**Published:** 2025-11-13

**Authors:** Zhongpeng Qiu, Dejing Shang

**Affiliations:** a School of Life Science, Liaoning Normal University, Dalian, 116081, China; b Liaoning Provincial Key Laboratory of Biotechnology and Drug Discovery, Liaoning Normal University, Dalian, 116081, China

**Keywords:** Type 2 diabetes mellitus, Pancreatic islet macrophages, β-cell, Insulin, Inflammation

## Abstract

The pathogenesis of type 2 diabetes mellitus (T2DM) is closely associated with chronic inflammation within the islet microenvironment, where pancreatic islet macrophages serve as central orchestrators of local immune regulation. This review provides a systematic overview of the ontogeny, phenotypic heterogeneity, and functional roles of pancreatic islet macrophages in T2DM pathology. Pancreatic islet macrophages contribute to β-cell proliferation and the maintenance of islet homeostasis through the secretion of various growth factors, such as platelet-derived growth factor (PDGF) and vascular endothelial growth factor (VEGF). Under conditions of metabolic stress, including lipotoxicity and glucotoxicity, these macrophages are polarized toward a pro-inflammatory phenotype. In this state, they impair β-cell function by releasing inflammatory mediators, including interleukin-1β (IL-1β) and tumor necrosis factor-α (TNF-α). Furthermore, this article discusses potential clinical strategies that target pancreatic islet macrophages—such as anti-inflammatory agents and immunomodulators—highlighting their promise as novel perspectives for precise intervention in T2DM.

## 1 Introduction

As a global public health challenge, T2DM currently affects more than 500 million individuals worldwide, with projections suggesting this number will exceed 800 million by 2050 [1]. This trend places a considerable burden on healthcare systems across the globe [2]. The core pathophysiological hallmarks of the disease include insulin resistance and a progressive decline in pancreatic β-cell function [3]. Conventional theories have attributed primarily β-cell dysfunction to peripheral metabolic disturbances, particularly obesity-associated lipotoxicity and hyperglycemia [[4], [5], [6]]. Over the past decade, chronic low-grade inflammation in the islet microenvironment has been recognized as a key mechanism driving T2DM progression [7,8]. Pancreatic islet macrophages, as central regulators of inflammatory responses, are highly sensitive to metabolic stress signals such as hyperglycemia and hyperlipidemia [9]. Through classical pathways involving Toll-like receptor 4 (TLR4) and NOD-like receptor family pyrin domain-containing 3 (NLRP3) inflammasome activation, these macrophages polarize toward the pro-inflammatory M1 phenotype [10,11]. Upon activation, M1 macrophages secrete large amounts of inflammatory mediators, IL-1β and TNF-α, which directly exert direct cytotoxic effects on β-cell and impair their glucose-stimulated insulin secretion capacity [12,13].

Clinical and pathological observations, demonstrate a critical role for pancreatic islet macrophages in the progressive decline of β-cell function [14]. In patients with T2DM, the degree of macrophage infiltration, as indicated by elevated CD68^+^ cell density, shows a positive correlation with the severity of β-cell dysfunction [15]. Animal models of T2DM further reveal that macrophage accumulation frequently occurs before the manifestation of overt hyperglycemia, implying that immune cell recruitment is an early event in disease pathogenesis [16,17]. Functional studies further support this notion. For example, macrophage depletion using clodronate liposomes improves pancreatic function and ameliorates insulin resistance [5]. Ongoing research into the activation, recruitment, and polarization mechanisms of pancreatic islet macrophages—particularly through signaling pathways such as chemokine CCL2 (also known as monocyte chemotactic protein-1 or MCP-1)/chemokine receptor 2 (CCR2), colony-stimulating factor 1 (CSF-1)/colony-stimulating factor 1 receptor (CSF-1R), and the NLRP3 inflammasome—has provided a rationale for targeted interventions [18]. Preclinical studies indicate that strategies employing CCR2 antagonists, CSF-1R inhibitors, or NLRP3 inhibitors hold promise for preserving β-cell function [19]. Recent technological progress in single-cell RNA sequencing (scRNA-seq) and spatial transcriptomics has uncovered the significant functional heterogeneity among pancreatic islet macrophages [15,20]. These approaches have also delineated their dynamic interactions with other islet cells, including β-cells [21]. Such high-resolution data provide novel perspectives on immune metabolic regulation within the islet and create a platform for designing precise, effective, and well-tolerated anti-inflammatory treatments [15].

The mouse model possesses intrinsic limitations for investigating the mechanisms underlying human diabetes. Currently, a substantial portion of mechanistic insights is derived from mouse models. While these models serve as valuable tools for elucidating disease pathogenesis, they represent, by nature, a simplified experimental system that falls short of fully recapitulating the considerable heterogeneity of human diabetes in terms of genetic background, immune features, and phenotypic manifestations. Moreover, gene knockout or transgenic studies conducted predominantly in a single inbred strain further constrain the generalizability of the resulting conclusions. Although this review systematically synthesizes research advances obtained from animal models, the translational relevance of these findings to human diabetes warrants careful evaluation to avoid overinterpretation or direct extrapolation from animal studies.

This review systematically examines the origin, heterogeneity, and functional properties of pancreatic islet macrophages, analyzes their polarization dynamics during T2DM progression, and assesses the translational potential of existing and emerging macrophage-targeted strategies. The aim of this review is to refine the theoretical framework for understanding immune-metabolic mechanisms in T2DM and facilitate the development of targeted treatment approaches.

### 1.1 Origin of pancreatic islet macrophages

Pancreatic islet macrophages are broadly classified by their origin and functional profiles into two main populations: islet-resident macrophages, which derive from embryonic yolk sac hematopoietic progenitors, and islet-recruiting macrophages, which originate from circulating monocytes in adulthood [22]. Moreover, the origin of these macrophages profoundly shapes their immune polarization state in the local tissue microenvironment, which in turn modulates β-cell function under both physiological and pathological conditions [23] (Table 1).

**Table 1:** Characteristics and functions of pancreatic islet macrophages.

**Feature Category**		**Islet-Resident Macrophages**	**Islet-Recruiting Macrophages**	Ref.
Organizational Area Room		inside the pancreas (in close contact with blood vessels)	intrapancreatic and peri pancreatic islets	[22,24,50]
Origin of Embryos		embryonic yolk sac	bone marrow mononuclear cells	[35]
Developmental Stage		the embryonic stage already exists, begins to mature at 1 week after birth, and reaches the adult phenotype at 4 weeks of age (in mice)	obesity or HFD starts to accumulate after 8 weeks, which is earlier than the onset of diabetes	[5,46]
Genealogy Markers	Mouse	F4/80, CD11b, CD206, CD301	F4/80, Ly6C, MHCII, CD68, CD11b, CD11c, CD206	[35,36,106]
	Human	CD163, CD204, CD206, CD301	CD68, CD11b, CD11c, CD163, CD204	[36,106]
Function		maintain pancreatic homeostasis	promote pancreatic inflammation	[42]
		immune surveillance	inhibit insulin secretion	[6,42]
		regulate metabolism	damage to β-cells	[14,94]
Turnover Maintenance		local proliferation self-renewal	recruiting circulating monocytes	[24,27]
Polarization Factor		IL-10, IL-4, IL-13, VEGF, M-CSF, ATP	High glucose, FFA, AGE, inflammatory cytokine, IAPP, ox-LDL	[[6], [7], [8]]
Logo Gene		Arg1, Fizz1, IL-10	IL-1β, TNF-α, IL-6	[[6], [7], [8]]
Metabolic Pathway		oxidative phosphorylation	glycolysis	[41]

**Fig. 1:**
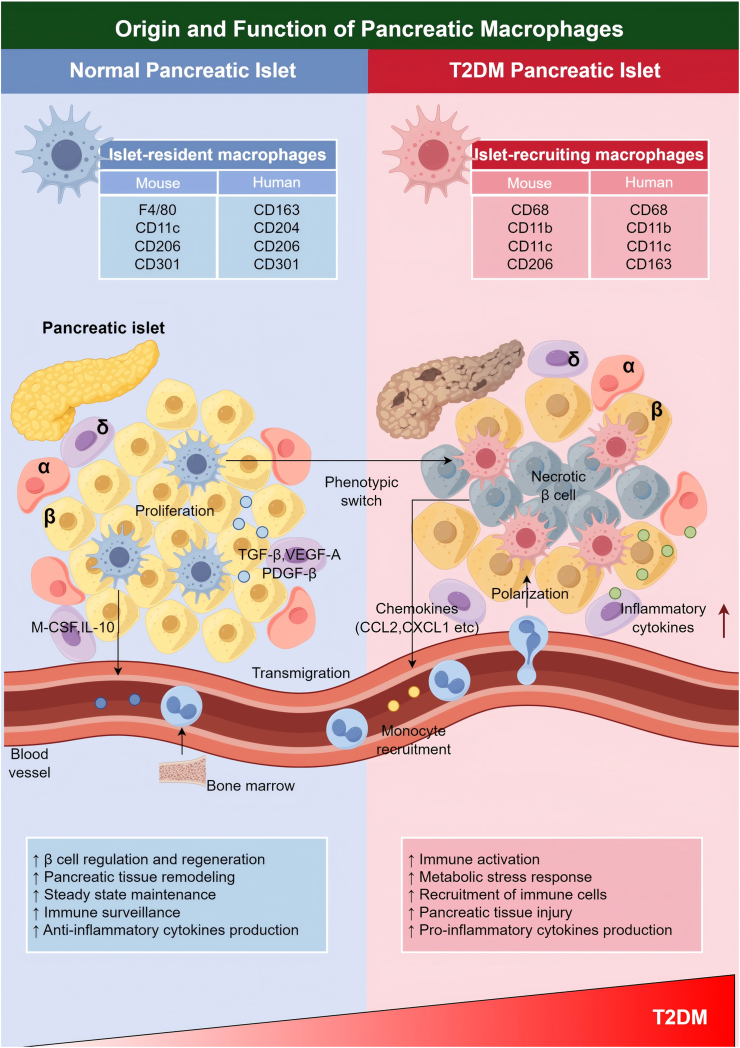
**Origin and Functional Heterogeneity of Pancreatic Islet Macrophages.** Pancreatic islet macrophages consist of two distinct populations: embryonically-derived tissue-resident macrophages and monocyte-derived macrophages recruited during adulthood, which display considerable heterogeneity in both developmental origin and functional properties. Tissue-resident macrophages of embryonic origin arise from early yolk sac hematopoietic progenitors, colonize the pancreatic primordia via primitive hematopoiesis, and contribute crucially to islet morphogenesis. These cells facilitate angiogenesis and promote β-cell proliferation through the secretion of factors such as M-CSF, IL-10, TGF-β, VEGF, and PDGF. In contrast, recruited macrophages are derived from Ly6C^+^ monocytes, which are mobilized to islets by chemokines including CCL2 and CXCL1 under metabolic stress conditions, such as obesity or high-fat diet feeding. These monocyte-derived macrophages typically adopt a pro-inflammatory phenotype, characterized by the production of cytokines like IL-1β and TNF-α, which contribute to the impairment of β-cell function. M-CSF: macrophage colony-stimulating factor; IL-10: interleukin-10; TGF-β: transforming growth factor-β; VEGF: vascular endothelial growth factor; PDGF: platelet-derived growth factor; CCL2: monocyte chemotactic protein-1; CXCL1: C-X-C motif chemokine ligand 1; IL-1β: interleukin-1β; TNF-α: tumor necrosis factor-α.

Islet-resident macrophages differentiate from yolk sac hematopoietic progenitor cells during early embryonic development and migrate to the pancreatic primordium via primitive hematopoietic processes, where they contribute to islet morphogenesis and functional maturation [24] (Fig. 1). Golden TN et al. reported that lineage tracing has revealed that these cells express F4/80^+^ between embryonic Days 8.5 and 14.5, and secrete growth factors such as VEGF and insulin-like growth factor (IGF) [25]. Through paracrine signaling, these factors support the development of the pancreatic vascular network and promote β-cell proliferation and differentiation [20]. Studies in macroph34age colony-stimulating factor (M-CSF)-deficient (Csf1op/op) mice have demonstrated that the absence of islet-resident macrophages results in pancreatic structural defects and reduced β-cell mass, underscoring their essential role in normal pancreatic development [26]. In adulthood, islet-resident macrophages primarily self-renew through local proliferation, maintaining a stable population with a long lifespan and a low turnover rate [27]. Epigenetically, these cells display an anti-inflammatory phenotype characterized by enrichment of histone H3K4me3 modifications at the promoters of anti-inflammatory genes [28]. This configuration sustains high expression of arginase-1 (Arg1) and the scavenger receptor 206 (CD206), and increases interleukin-10 (IL-10) secretion, enabling effective clearance of apoptotic cells and maintenance of immune homeostasis in the pancreas [29] (Table 1).

Islet-recruiting macrophages derive predominantly from circulating monocytes in adults [30]. Under metabolic stress conditions such as obesity, hyperglycemia, or hyperlipidemia, they are recruited in large numbers to the pancreas via chemokine signaling [31]. As shown in Fig. 1, central to this process is the interaction between CCL2 and its receptor CCR2 [24]. Elevated glucose and free fatty acids (FFA) stimulate islet cells to secrete CCL2, enhancing monocyte adhesion and migration across the endothelium into the pancreas [7] (Table 2). Additional chemokines, including CXCL1, CCL3, and CCL5, also participate in monocyte recruitment [32]. In the pancreatic microenvironment, monocytes differentiate into pro-inflammatory M1-type macrophages under the influence of high levels of glucose, lipids, and inflammatory cytokines [7]. These cells express surface markers such as scavenger receptor 11c (CD11c) and scavenger receptor 68 (CD68), and secrete pro-inflammatory mediators such as TNF-α and IL-1β [33]. Furthermore, West well-Roper C et al. discovered that they facilitate IL-1β maturation and release via NLRP3 inflammasome activation, amplifying local inflammation and promoting β-cell apoptosis and pancreatic fibrosis [34]. In T2DM models, such as leptin receptor deficiency (db/db) mice and high-fat diet (HFD)-induced obese mice, the abundance of monocyte-derived pancreatic islet macrophages increases markedly, indicating that these cells are key drivers of islet inflammation and β-cell dysfunction [5,16]. Metabolic stressors such as FFA and advanced glycation end product (AGE) can further activate Toll-like receptor (TLR) signaling—particularly TLR4—in these macrophages, leading to nuclear factor kappa B (NF-κB) and mitogen-activated protein kinase (MAPK) pathway activation, which exacerbates the inflammatory response [34]. This recruitment mechanism not only aggravates pancreatic pathology but also establishes a positive feedback loop that sustains chronic inflammation through continuous immune cell infiltration [7].

**Table 2:** Supporting evidence for CCL2-CCR2 dependent recruitment amplifying inflammation.

Condition	Mouse	Human	Ref.
Duration of Diet	8-week HFD: early accumulation of CD11b^+^ islet macrophages	pre diabetes: inflammatory markers have increased, but the number of macrophages has not reached the T2DM level	[16,17,53]

long term HFD (>12 weeks): pro-inflammatory M1 macrophages dominate, replacing resident M2 macrophages	obese individuals: IL-1 β, TNF - α, and IL-6 in pancreatic islets are significantly elevated, and macrophage phenotype is pro-inflammatory	[3,5,6]

Disease Stage	pre diabetes (8 weeks of HFD): peripheral macrophages begin to infiltrate around the islets	pre diabetes: M1 polarization intensifies	[4,5,16]
	late stage of T2DM: M1 macrophages account for more than 60 %, with high expression of pro-inflammatory cytokines	late stage of T2DM: increased number of CD68^+^ islet macrophages	[15,16]
Model Strain	C57BL/6: The recruited islet macrophages are supplemented by monocytes recruited by CCR2, but the islet-resident macrophages are independent of CCR2	healthy individuals: islet macrophages maintain homeostasis, with low expression of CCL2	[30,31]
	db/db: M1 macrophage amplification in pancreatic islets depends on CCL2	T2DM patients: CCL2 expression is positively correlated with islet macrophage density	[24,27]
Gender	HFD induced islet macrophage infiltration is more severe in male mice	**-**	[27]
Age	aging mouse islet macrophage are not associated with CCR2	islet macrophages in aging population are not related to CCL2	[24,112]
Relative Abundance	steady state: islet-resident macrophages account for>90 %	steady state: dominated by islet-resident macrophages	[22,24]
	T2DM: islet-recruiting macrophage account for 70 %, and resident cells decrease to<30 %	T2DM: islet-recruiting macrophage account for 60–80 %	[30,113]

### 1.2 Heterogeneity of pancreatic islet macrophages

The heterogeneity of pancreatic islet macrophages is primarily defined by their distinct developmental origins, spatial distributions, functional properties, and metabolic profiles. Two main subsets can be distinguished: those arising from embryonic precursors and those derived from adult bone marrow [35]. Under steady-state conditions, embryonic-derived islet-resident macrophages represent the predominant population [35]. These cells are long-lived and capable of local self-renewal. In contrast, bone marrow-derived macrophages originate from circulating Ly6C^+^/CCR2^+^ monocytes that are recruited during inflammation or metabolic disturbance, and they generally exhibit greater plasticity and a shorter tissue residence time [27].

The differential origins result in fundamental distinctions in the spatial localization and physiological roles of the two subsets within the pancreatic microenvironment [24]. Embryonic-derived islet-resident macrophages are predominantly located in proximity to β-cell, where they play a pivotal role in supporting β-cell proliferation and modulating local angiogenesis through synaptic-like contacts and the secretion of factors such as IGF and transforming growth factor β (TGF-β) [22,36]. Their phenotype is characterized as F4/80^+^ CD11b^+^ CD11c^−^ CD206^+^, reflecting M2-like anti-inflammatory features under homeostasis [35]. Conversely, islet-recruiting macrophages are frequently localized at the interface between islets and exocrine tissue, often adjacent to blood vessels. They typically express F4/80^+^ CD11b^+^ CD11c^+^ CD68^+^ and are involved primarily in immune surveillance and the subsequent mediation of monocyte recruitment under metabolic stress [16,37] (Fig. 1).

Beyond their developmental origin and tissue distribution, pancreatic islet macrophages also demonstrate distinct polarization states and functional attributes. Under physiological conditions, embryonic-derived islet-resident macrophages generally maintain an anti-inflammatory M2 phenotype, which is sustained by signaling molecules such as interleukin-4 (IL-4), interleukin-13 (IL-13), IL-10, and CSF-1 [38]. M2-polarized macrophages express markers such as CD206 and Arg1, and secrete high levels of IL-10 and TGF-β, thereby fostering a microenvironment conducive to pancreatic homeostasis and enhancing β-cell insulin secretion [20,21] (Table 1). In contrast, under the pathological conditions of T2DM, factors such as hyperglycemia, hyperlipidemia, endotoxins such as lipopolysaccharides (LPS), and pro-inflammatory cytokines promote the polarization of islet-recruiting macrophage toward the classical pro-inflammatory M1 phenotype [39]. Activated M1 macrophages upregulate CD11c and inducible nitric oxide synthase (iNOS) expression, and release mediators such as IL-1β, TNF-α, and reactive oxygen species (ROS), contributing to β-cell dysfunction and apoptosis [40].

Substantial differences in metabolic characteristics also exist between these Pancreatic Islet Macrophage subsets. ScRNA-seq revealed that islet-resident macrophages predominantly utilize fatty acid oxidation and oxidative phosphorylation [41]. These are metabolic pathways that support their anti-inflammatory functions. In contrast, islet-recruiting macrophages upregulate genes associated with inflammatory pathways and undergo metabolic reprogramming toward glycolysis to meet energy demands for rapid activation [41]. This metabolic divergence not only reflects their distinct origins and differentiation pathways but also underlies their opposing roles in physiology and disease. Specifically islet-resident macrophages help maintain pancreatic homeostasis, whereas islet-recruiting macrophages promote inflammation and tissue injury [42].

### 1.3 Functions of pancreatic islet macrophages

#### 1.3.1 Islet-resident macrophages

**Fig. 2:**
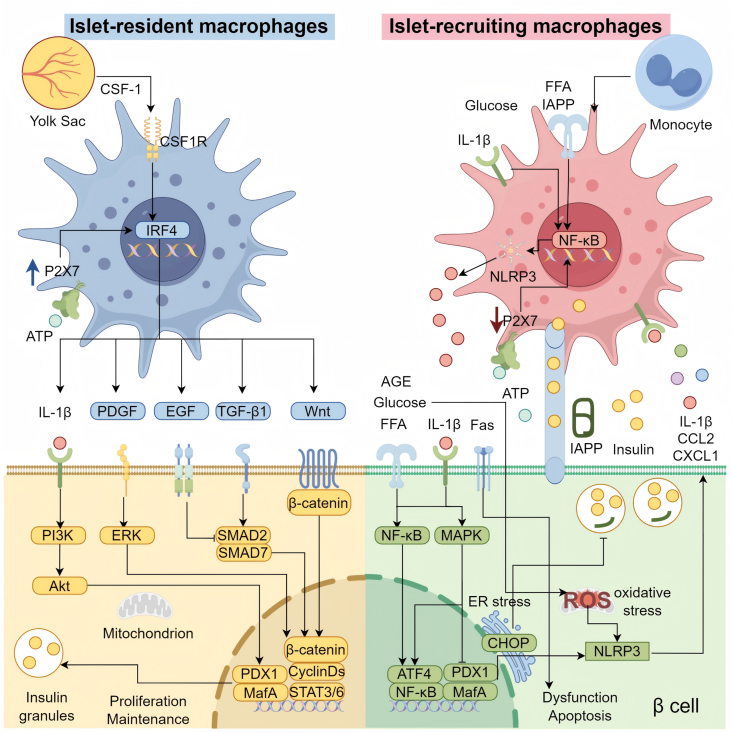
**Crosstalk between Pancreatic Islet Macrophages and β-cells.** Under the influence of CSF-1, embryonic yolk sac-derived precursors differentiate into islet-resident macrophages. These macrophages facilitate the expression of anti-inflammatory genes via transcriptional regulators such as IRF4. Islet-resident macrophages maintain phenotypic stability by sensing β-cell-derived ATP through purinergic receptors, including P2X7, thereby dynamically modulating IL-1β release to align with insulin secretory demands. Furthermore, islet-resident macrophages secrete factors such as PDGF, EGF, TGF-β, and Wnt, which influence the activity of transcription factors including PDX1, MafA, and β-catenin via ERK, SMAD, and Wnt signaling pathways, thereby promoting β-cell proliferation and insulin secretion. Islet-recruiting macrophages originate primarily from circulating monocytes. Stimulated by factors such as FFA, IAPP, high glucose, and IL-1β, these macrophages activate the NLRP3 inflammasome and release proinflammatory cytokines including IL-1β through NF-κB signaling. These cytokines suppress the transcription of key β-cell insulin genes, such as PDX1 and MafA, and impair insulin secretion. Additionally, FFA, IAPP, and high glucose exacerbate β-cell dysfunction via NF-κB, MAPK pathways, oxidative stress, and endoplasmic reticulum stress, further promoting NLRP3 inflammasome activation and the secretion of IL-1β and chemokines such as CCL2. These inflammatory mediators create a feedback loop that amplifies macrophage recruitment and activation. Islet-recruiting macrophages also induce β-cell apoptosis via the Fas/FasL pathway and directly deplete insulin granules by uptaking insulin vesicles through TNT. In obesity, downregulation of purinergic receptors (e.g., P2X7) on macrophages leads to ATP signaling desensitization, attenuates β-cell stress responses, and contributes to insulin secretory deficiency.CSF-1: colony-stimulating factor 1; IRF4: Interferon Regulatory Factor 4; ATP: adenosine triphosphate; IL-1β: interleukin-1β; PDGF: platelet-derived growth factor; EGF: epidermal growth factor; TGF-β: transforming growth factor β; ERK: Extracellular Regulated Kinases; SMAD: SMA and MAD homologs; PDX1: pancreatic duodenal homeobox factor-1; MafA: Musculoaponeurotic fibrosarcoma oncogene homolog A; FFA: free fatty acids; IAPP: islet amyloid polypeptide; NF-κB: nuclear factor kappa B; NLRP3: NOD-like receptor family pyrin domain-containing 3; MAPK: mitogen-activated protein kinase; CCL2: monocyte chemotactic protein-1; Fas: Fas cell surface death receptor; FasL: Fas ligand; TNT: tunneling nanotube.

As key regulators of the pancreatic microenvironment, pancreatic islet macrophages critically influence β-cell development, cellular protection, homeostasis maintenance, and intercellular communication via cytokine secretion, metabolic modulation, and signaling networks (Fig. 2).

During pancreatic embryogenesis, islet-resident macrophages contribute to β-cell differentiation and islet morphogenesis through multiple mechanisms. As shown in Fig. 2, these macrophages promote β-cell generation by secreting CSF-1 and M-CSF, facilitating the exit of pancreatic epithelial cells from the cell cycle and their migration toward endocrine differentiation zones [43]. Furthermore, macrophages accumulate at pancreatic duct branches and support the formation and remodeling of local vascular networks through the secretion of angiogenic factors such as VEGF [44]. This neovascularization supplies developing islets with oxygen and nutrients, while also transporting endocrine precursor cells and potentially directly influencing β-cell differentiation and maturation via vascular endocrine signals [20]. The pancreatic duodenal homeobox factor-1 (PDX1) serves as a core transcription factor in pancreatic development and β-cell function, with its expression critically influencing β-cell fate and functional maturation [3]. Studies also indicate that pancreatic islet macrophages enhance the proliferation and differentiation of β-cell progenitors such as PDX1^+^ cells through the production of Arg1 and polyamines [45].

Islet-resident macrophages employ diverse dynamic regulatory mechanisms to maintain homeostasis and immune surveillance. Ultrastructural analyses revealed that macrophage bodies extend filamentous pseudopods into the vascular lumen, increasing their surface area for sensing circulating metabolic signals such as FFA and LPS [46]. They also highly express the purinergic receptor P2X7. Upon glucose-stimulated insulin secretion, β-cell release adenosine triphosphate (ATP), which is sensed by P2X7 [47]. This interaction triggers downstream signaling and fine-tunes the release of cytokines such as IL-1β [48]. This response is not purely pro-inflammatory but operates within a physiological range to support insulin secretory feedback. Macrophages and β-cell may also establish synapse-like connections, enabling direct uptake and processing of insulin granules via vesicular mechanisms, which may modulate insulin secretion or facilitate β-cell antigen presentation [9,46]. In addition to having sensory and secretory effects, pancreatic islet macrophages serve as efficient scavengers, clearing apoptotic β-cell and debris through potent phagocytosis to prevent secondary inflammation caused by damage-associated molecular patterns (DAMPs) [49]. Concurrently, they release immunomodulatory and reparative factors such as IL-10 and TGF-β, which help sustain local immune tolerance [50].

In the context of β-cell protection and regeneration, pancreatic islet macrophages display polarizable immunoregulatory properties. In injury models such as those involving partial pancreatic duct ligation, these cells adopt an M2-like phenotype and secrete factors such as IL-10, TGF-β, and PDGF [18]. This phenotypic shift suppresses NF-κB-mediated inflammation and enhances β-cell survival via Phosphatidylinositol 3-kinase (PI3K)/Akt (also known as phosphatidylinositol-dependent kinase B or PKB) activation [51]. Akt phosphorylated proapoptotic proteins such as Fork head box O1 (FoxO1), and upregulates antiapoptotic factors such as B-cell lymphoma/leukemia-2‌ (Bcl-2), thereby increasing resistance to stress-induced apoptosis [52]. Beyond direct cellular protection, islet-resident macrophages also promote β-cell regeneration through secreted factors such as PDGF and epidermal growth factor (EGF) [18]. These factors activate proliferative pathways such as ‌Extracellular Regulated Kinases (ERK)/MAPK to drive β-cell replication and survival.

#### 1.3.2 Islet-recruiting macrophages

In the pathogenesis of T2DM, pancreatic islet macrophages serve as core effector cells that contribute to β-cell dysfunction [37] (Fig. 2). Under physiological conditions, monocytes circulate in the bloodstream and patrol tissue microenvironments [4]. However, under metabolic stress such as hyperglycemia and hyperlipidemia, they are recruited in large numbers to the pancreas and polarize into a pro-inflammatory M1 phenotype, thereby driving inflammatory responses [12]. These macrophages primarily activate inflammation via the TLR4/NLRP3 inflammasome signaling pathway [34]. This pathway activates the myeloid differentiation factor 88 (MyD88)-dependent cascade to initiate NF-κB signaling, which promotes the transcription and secretion of pro-inflammatory cytokines including IL-1β, TNF-α, and interleukin-6 (IL-6) [53]. Such cytokines not only directly suppress insulin secretion from β-cell but also disrupt insulin signaling, establishing a pathogenic feedback loop [54].

Direct contact between macrophages and β-cell further aggravates cellular injury. By tunneling nanotubes (TNTs), M1 macrophages internalize insulin secretory granules from β-cell [51]. This results in the disruption of calcium signaling and ATP depletion, which severely compromising glucose-stimulated insulin secretion (GSIS) [55]. This interaction is accompanied by the release of chemokines such as CXCL10, recruiting additional macrophages to the pancreas and perpetuating a self-sustaining inflammatory cascade [51]. Under glucolipotoxic conditions, macrophages undergo marked metabolic reprogramming. Mitochondrial oxidative phosphorylation is impaired, whereas glycolysis is increased, leading to the accumulation of lactate and ROS [41]. This metabolic shift activates Hypoxia-inducible factor 1-alpha (HIF-1α), upregulating the expression of pro-inflammatory genes including IL-1β and TNF-α, and further suppressing mitochondrial function [41].

Macrophage-derived cytokines impair β-cell function and survival through multiple molecular pathways. IL-1β downregulates key β-cell transcription factors such as PDX1 and Musculoaponeurotic fibrosarcoma oncogene homolog A (MafA), and induces endoplasmic reticulum (ER) stress and mitochondrial dysfunction via c-Jun N-terminal kinase (JNK) and NF-κB signaling, thereby inhibiting proinsulin processing [13]. Under high-glucose conditions, Li L et al. discovered that macrophage-secreted TNF-α suppresses the expression of the mitochondrial fusion protein 2 (MFN2), disrupts metabolic-secretory coupling in β-cell, and ultimately promotes β-cell dedifferentiation and functional impairment [56]. Furthermore, macrophages and β-cell engage in a self-amplifying inflammatory circuit. Specifically, Wang G et al. indicated that IL-1β induces cyclooxygenase-2 (COX-2) expression in β-cell, promoting prostaglandin E_2_ (PGE_2_) production, which not only inhibits insulin secretion-related genes but also stimulates further IL-1β release from macrophages via prostaglandin E_2_ receptor (EP3R) signaling [57]. Studies also indicate that macrophages activated by human islet amyloid polypeptide‌ (hIAPP) significantly suppress insulin gene expression and GSIS in conditioned medium, implicating amyloid polypeptides in this pathological process [34]. Collectively, these mechanisms lead to a decrease in β-cell function, characterized by suppressed insulin gene expression, impaired proinsulin processing, and a loss of glucose sensitivity.

### 1.4 Pancreatic islet macrophages in T2DM

**Fig. 3:**
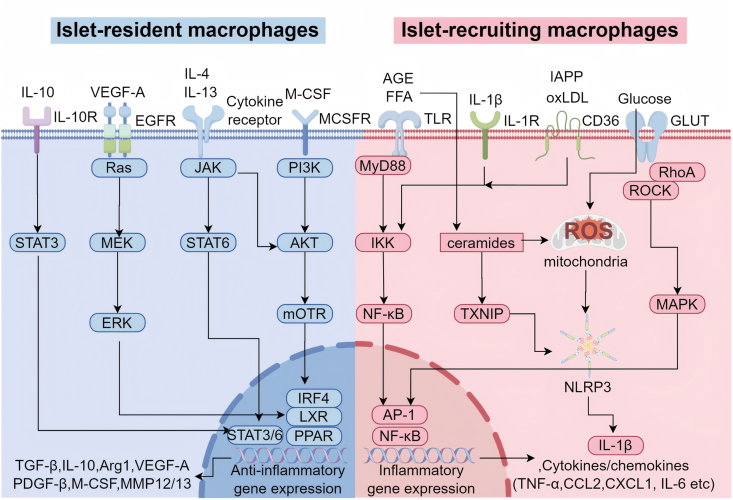
**The Polarization Mechanism of Pancreatic Islet Macrophages Induced by Metabolic Stress.** Islet-resident macrophages sense circulating factors such as IL-4, IL-10, and IL-13, and maintain their phenotype via the STAT3/STAT6 signaling pathway. Under stimulation by VEGF and M-CSF, these macrophages modulate transcription factors including IRF4 and PPAR-γ through ERK, PI3K, and Akt signaling, thereby enhancing the expression of anti-inflammatory genes such as TGF-β, IL-10, and Arg1. Islet-recruiting macrophages, in response to metabolic stressors—such as AGEs, FFAs, high glucose, ox-LDL, and IAPP—activate the TLR4/MyD88–NF-κB signaling axis and the NLRP3 inflammasome, leading to the production of pro-inflammatory cytokines including IL-1β and TNF-α, as well as chemokines like CCL2. This process establishes a vicious cycle termed “metabolic inflammation,” which involves ROS generation, ultimately contributing to beta cell failure.IL-4: interleukin-4; IL-10: interleukin-10; IL-13: interleukin-13; STAT3/6: signal transducer and activator of transcription 3/6; VEGF: vascular endothelial growth factor; M-CSF: macrophage colony-stimulating factor; ERK: Extracellular Regulated Kinases; PI3K: Phosphatidylinositol 3-kinase; Akt: phosphatidylinositol-dependent kinase B; IRF4: Interferon Regulatory Factor 4; PPAR-γ: peroxisome proliferator-activated receptor γ; TGF-β: transforming growth factor β; Arg1: arginase-1; AGE: advanced glycation end product; FFA: free fatty acids; ox-LDL: Oxidized low-density lipoprotein; IAPP: islet amyloid polypeptide; TLR4: Toll-like receptor 4; MyD88: myeloid differentiation factor 88; NF-κB: nuclear factor kappa B; NLRP3: NOD-like receptor family pyrin domain-containing 3; IL-1β: interleukin-1β; TNF-α: tumor necrosis factor α; CCL2: monocyte chemotactic protein-1; ROS: reactive oxygen species.

In T2DM, phenotypic alterations and dysfunction of pancreatic islet macrophages represent central pathological mechanisms that drive islet inflammation and impair β-cell function. Under physiological conditions, pancreatic islet macrophages consist primarily of yolk sac–derived resident populations and bone marrow–derived recruited subsets, which exist in a homeostatic balance to maintain normal pancreatic function and immune regulation [58]. Under conditions of metabolic stress, however, this equilibrium is disrupted, leading to a marked increase in the abundance and activity of islet-recruiting macrophages [4]. Moreover, the anti-inflammatory capacity of islet-resident macrophages is suppressed. As illustrated in Fig. 3, metabolic stressors such as high glucose and FFA activate the TLR4/NF-κB signaling pathway, promoting macrophage polarization toward the pro-inflammatory M1 phenotype [53]. These M1 macrophages are characterized by elevated secretion of inflammatory mediators including IL-1β, TNF-α, and nitric oxide (NO) [53]. Furthermore, pathological aggregation of islet amyloid polypeptide (IAPP) activates the NLRP3 inflammasome, facilitating the maturation and release of IL-1β and thereby establishing a self-amplifying inflammatory feedback loop [59].

#### 1.4.1 High glucose

In the pathogenesis of T2DM, sustained hyperglycemia acts as a critical metabolic stressor that promotes the polarization of islet-associated macrophages toward a pro-inflammatory M1 phenotype.

Hyperglycemia can directly engage pattern recognition receptors on macrophages, initiating downstream inflammatory signaling cascades (Fig. 3). Studies have indicated that under hyperglycemic conditions, TLR4 expression is markedly upregulated in macrophages [60]. This leads to NF-κB activation via the MyD88-dependent pathway and subsequent transcription and secretion of pro-inflammatory cytokines such as IL-1β, TNF-α, and IL-6. Additionally, hyperglycemia-induced mitochondrial ROS overproduction facilitates NLRP3 inflammasome activation, resulting in caspase-1-dependent maturation and the release of IL-1β [61].

ER stress represents another key mechanism through which hyperglycemia drives M1 polarization. Glucose fluctuations activate the inositol-requiring enzyme 1α (IRE1α)–X-box binding protein 1 (XBP1) signaling axis, upregulating the expression of ER chaperones [62]. This cascade enhances activator protein 1 (AP-1) transcriptional activity, thereby amplifying the production of pro-inflammatory cytokines. In mouse models of db/db and diet induced obesity (DIO), Morikawa S et al. reported that pancreatic islet macrophages exhibit significant IRE1α overactivation, increased XBP1 splicing, and upregulated expression of inflammatory genes [40].

Furthermore, hyperglycemia reinforces macrophage inflammatory function by driving metabolic reprogramming. The recruitment of monocytes to pancreatic islets increases glycolytic flux via glucose transporter type 1‌ (GLUT1)-mediated glucose uptake, the stabilization of HIF-1α and the induction of the expression of IL-1β and IL-6 [41].

Multiple protein kinase pathways collectively mediate hyperglycemia-induced macrophage polarization. Under hyperglycemic conditions, protein kinase C (PKC) activation promotes the phosphorylation of signal transducer and activator of transcription 1‌ (STAT1) [33]. This increases its transcriptional activity and facilitates the expression of M1-polarizing regulators such as interferon regulatory factor 5 (IRF5) and interferon regulatory factor 8 (IRF8) [5].

#### 1.4.2 High fat

In the pathogenesis of T2DM, FFA act as critical lipotoxic mediators that promote the polarization of pancreatic islet macrophages toward a pro-inflammatory M1 phenotype through the activation of specific receptor signaling pathways and the induction of metabolic reprogramming. This process promotes a chronic inflammatory microenvironment that markedly accelerates β-cell dysfunction (Fig. 3).

Palmitate (PA) can directly bind to and activate TLR4 on the macrophage surface, triggering downstream inflammatory signaling cascades [39]. TLR4 activation leads to the recruitment of the adaptor protein MyD88 via its intracellular Toll/interleukin-1 receptor (TIR) domain. MyD88 subsequently activates the IL-1 receptor-associated kinase (IRAK) complex, resulting in the phosphorylation of TNF receptor-associated factor 6 (TRAF6) and the subsequent activation of transforming growth factor-β-activated kinase 1 (TAK1) [63]. These events ultimately drive the activation of NF-κB and MAPK pathways, upregulating the expression of chemokines and cytokines such as IL-1β, TNF-α, CCL2, and CXCL1 (Table 2).

Following cellular uptake, FFA are metabolized into lipophilic intermediates that further potentiate inflammatory signaling. When internalized via the scavenger receptor 36 (CD36), FFA can be converted into ceramides, which activate PKC and JNK, thereby reinforcing NF-κB signaling [64]. Studies in mice have shown that HFD feeding upregulates TLR4 expression in pancreatic islet macrophages, whereas TLR4 knockout abrogates FFA-induced IL-1β secretion and insulin resistance [53]. In addition, a HFD induces the accumulation of saturated fatty acids within the pancreas, resulting in mitochondrial dysfunction and the release of mitochondrial DNA (mtDNA) into the cytosol [65]. Hong Z et al. showed that the leaked mtDNA subsequently activates the cyclic GMP-AMP synthase (cGAS)–stimulator of interferon genes (STING) signaling pathway in macrophages, thereby further amplifying the inflammatory response.

Lipid overload induces ER stress, activating all three branches of the unfolded protein response (UPR). Excess lipids disrupt intracellular calcium homeostasis, leading to activation of the IRE1α–XBP1, protein kinase R-like endoplasmic reticulum kinase (PERK)–eukaryotic translation initiation factor 2 subunit α (eIF2α), and activating transcription factor 6 (ATF6) pathways [66]. Furthermore, Wang S-W et al. demonstrated that ER stress and oxidative stress cooperatively promote NLRP3 inflammasome activation and IL-1β maturation. C/EBP homologous protein (CHOP)-induced thioredoxin-interacting protein (TXNIP) binds to and inhibits thioredoxin (TRX), increasing mitochondrial reactive oxygen species (mtROS) production [67].

#### 1.4.3 Islet amyloid

The abnormal aggregation of IAPP represents a core pathological hallmark of pancreatic islets in T2DM, where it sustains inflammatory responses through specific activation of islet-resident macrophages [68] (Fig. 3). Under pathological conditions, IAPP monomers secreted by β-cell can assemble into oligomers enriched in β-sheet structures, which act as critical triggers for innate immune activation [34]. Pancreatic islet macrophages specifically detect these IAPP oligomers via pattern recognition receptors, initiating downstream inflammatory signaling [34].

In particular, IAPP oligomers are recognized by TLR2/4 as well as the CD36 on macrophages, leading to the activation of a MyD88-dependent signaling pathway [69]. This pathway promotes the activation of NF-κB and MAPK cascades. This drives the transcription of pro-inflammatory cytokines such as TNF-α and IL-6, and facilitating NLRP3 inflammasome assembly [70]. Furthermore, amyloid deposition disrupts macrophage autophagic flux, impairs the clearance of hIAPP, and promotes the accumulation of intracellular aggregate, thereby exacerbating disruption of cellular homeostasis [71]. Using a hIAPP transgenic mouse model, West well-Roper CY et al. demonstrated that IAPP aggregates induce a phenotypic shift in islet-resident macrophages, characterized by elevated expression of CD11b and CD11c, ultimately resulting in pancreatic dysfunction [72].

#### 1.4.4 Oxidized low-density lipoprotein

Oxidized low-density lipoprotein (ox-LDL) is a significant pathogenic factor in the islet microenvironment of T2DM, exacerbating islet inflammation and impairing β-cell function through multiple mechanisms. Pancreatic islet macrophages recognize and take up ox-LDL via specific scavenger receptors, resulting in intracellular lipid accumulation. ox-LDL is specifically recognized and bound by scavenger receptors such as CD36 and lectin-like oxidized low-density lipoprotein receptor 1 (LOX-1) on the macrophage surface, leading to substantial intracellular lipid deposition and eventual foam cell formation [73]. This constitutes an important basis for establishing a lipotoxic islet microenvironment. This subsequently activates the NF-κB signaling pathway and upregulates the expression of pro-inflammatory cytokines IL-1β, TNF-α, and IL-6 [73]. These findings were further corroborated in RAW264.7 macrophages exposed to ox-LDL, which subsequently displayed elevated ROS production, enhanced caspase-1 activity, and increased secretion of IL-1β [74]. Srivastava N et al. reported that pancreatic islet macrophages help maintain pancreatic microenvironment homeostasis via CXCL16-mediated clearance of ox-LDL. In NOD mice, loss of CXCL16 leads to ox-LDL accumulation, which compromises the survival and effector function of infiltrating CD8^+^ T cells in pancreatic islets, thereby delaying diabetes onset [75].

#### 1.4.5 Advanced glycation end products

AGE serve as key inflammatory mediators within the islet microenvironment in T2DM. They induce macrophage polarization toward a pro-inflammatory phenotype primarily through receptor-mediated signaling pathways. The interaction between AGE and their specific receptor, receptor for advanced glycation end products (RAGE), initiates downstream inflammatory signaling cascades [49]. He S et al. demonstrated that this AGE-RAGE interaction effectively activates the ROS-MAPK-NF-κB signaling axis [76]. In vitro studies indicate that stimulation with AGE markedly upregulates the transcription of pro-inflammatory cytokines in macrophages [49]. This leads to MAPK pathway activation and increased transcription of pro-inflammatory cytokines, including IL-1β, TNF-α, and IL-6.

The AGE-RAGE axis can synergize with other pattern recognition receptors to amplify inflammatory responses. AGE signaling upregulates TLR4 expression on macrophages, thereby establishing a synergistic RAGE-TLR4 axis [11]. Compared with untreated macrophages, Liu Z et al. discovered that macrophages pretreated with AGE exhibit elevated TLR4 levels and an increased inflammatory response to subsequent H_2_O_2_ challenge [11]. Furthermore, AGE sustain macrophage inflammation by influencing metabolic reprogramming. Han X et al. indicated that this role was confirmed in pyruvate dehydrogenase kinase 4‌ (PDK4)-deficient macrophages, in which loss of PDK4 resulted in significantly reduced IL-1β secretion upon AGE exposure, underscoring the importance of metabolic reprogramming in maintaining the inflammatory phenotype driven by AGE [77].

Beyond these mechanisms, various pathological stimuli also promote Pancreatic Islet Macrophage polarization through crosstalk signaling.

### 1.5 Targeted treatment strategies for pancreatic islet macrophages

The inhibition of pro-inflammatory polarization in pancreatic islet macrophages represents a central therapeutic strategy for ameliorating islet inflammation in patients with T2DM (Table 3). Preclinical evidence indicates that the TLR4 antagonist TAK-242 suppresses AGE-induced activation of the STAT1 pathway, thereby reducing IL-1β secretion in cultured macrophages [78]. In NOD mice, Alibashe-Ahmed M et al. discovered that treatment with TAK-242 markedly attenuated islet inflammation, improved glucose tolerance and insulin secretion, and prevented the onset of diabetes [79]. Targeting AGE with a RAGE monoclonal antibody has been demonstrated to effectively inhibit AGE–RAGE interaction and alleviate chronic inflammation in diabetic mice [80]. In addition, in vitro studies revealed that the Rho kinase inhibitor Fasudil ameliorated macrophage infiltration in diabetic rats, increased the abundance of M2 macrophages and anti-inflammatory cytokine levels, and suppressed the progression of diabetes, obesity, and dyslipidemia [81]. The NLRP3 inflammasome plays a critical role in pancreatitis by promoting caspase-1-dependent maturation and release of IL-1β [10]. Preclinical studies have confirmed that MCC950, an NLRP3 inflammasome inhibitor, increases M2 macrophage polarization and reduces pro-inflammatory gene expression in diabetic mice by inhibiting NLRP3 activation [82]. Wang J-L et al. reported that the G protein-coupled receptor 132 (GPR132) antagonist NOX-6-18 effectively blocks its endogenous ligand 9(S)-HODE-induced Gi signaling, thereby modulating macrophage reprogramming in the islets of HFD-fed mice, attenuating weight gain, and improving glucose metabolism [83].

**Table 3:** Drugs targeting pancreatic islet macrophages.

Intervention Targets/Drugs	Main Mechanism	Main Phenomena	Duration	Development Phase	Human Evidence	Main Risks	Ref.
NLRP3 inhibitors (MCC950)	inhibit NLRP3 inflammasome assembly, block IL-1 β maturation and release	pancreatic inflammation ↓; β-cell function ↑; blood sugar ↓	continue until discontinuation (animal study)	preclinical	Inhibition of inflammasomes can reduce apoptosis of human β-cells	may weaken innate immune defense, and Liver toxicity	[10,82]
IL-1 β antagonists (Canakinumab)	specifically neutralizing IL-1 β and inhibiting inflammasome activation	insulin secretion ↑; pro-inflammatory factors ↓	weeks to months (clinical trial)	II/III	cardiovascular benefits, but limited improvement in blood glucose	increased risk of infection; may weaken wound healing	[94]
CSF-1R inhibitors (PLX3397)	inhibit macrophage proliferation and survival, reduce pancreatic M1 macrophage infiltration	pancreatic inflammation ↓; β-cell function ↑	long term treatment is effective	preclinical	preclinical evidence: Exhausting macrophages can reverse human pancreatic inflammation	bone marrow suppression (neutropenia), liver toxicity, potential tissue repair disorders	[114]
GPR132 antagonist (NOX-6-18)	blocking GPR132 and inhibiting macrophage polarization towards pro-inflammatory phenotype	pro-inflammatory factors ↓; insulin resistance ↓	single administration can last for 24 h	preclinical	preclinical evidence: GPR132 mediates lipid inflammatory signaling in human macrophages	long term inhibition of GPCR signaling may affect macrophage homeostasis function	[115]
TLR4 antagonists (TAK-242)	inhibition of MyD88 dependent inflammatory pathway	inflammatory cytokines (IL-1 β, TNF - α) ↓; pancreatic inflammation ↓	single dose administration (clinical)	II	NCT02705833: Not reaching the primary endpoint	may increase the risk of infection	[11,78]
FABP4 inhibitors (BMS309403)	blocking fatty acid transport and inhibiting macrophage inflammatory response	IL-1 β release ↓; insulin resistance ↓	several hours	preclinical	**-**	may affect fat metabolism	[79]
Rho kinase (ROCK) inhibitors (Fasudil)	inhibit macrophage migration and inflammatory cytokine release	pancreatic microcirculation ↑; macrophage infiltration ↓	short term (acute phase treatment)	II	J-ROCK study: reduces urinary protein in T2DM patients, but does not significantly improve blood glucose levels	low blood pressure risk, may affect wound healing	[81]
RAGE monoclonal antibody	blocking the receptor for RAGE and inhibiting NF - κ B activation	macrophage activation ↓; β-cell apoptosis ↓	weeks to months	II	NCT00758029: Limited efficacy and acceptable safety in T2DM patients	immunogenic risk, may affect tissue repair	[80,116]
endogenous histone deacetylase (HDAC) inhibitors (β - hydroxybutyrate)	inducing anti-inflammatory phenotype of macrophages	pancreatic inflammation ↓; IL-1β↓	blood ketone concentration	preclinical	small study: Ketogenic diet improves blood glucose in T2DM patients	long term hyperketosis or acidosis; risk of nutritional imbalance	[92,93]
Vitamin D3	combining VDR nuclear receptors to inhibit macrophage TNF - α/IL-6 production	HOMA-IR↑	long term supplementation	approve	Meta analysis: Improving insulin sensitivity	risk of hypercalcemia	[85,117]
IL-4/IL-13 mRNA liposomes	LNP delivers mRNA encoding IL-4/IL-13, local expression induces M2 polarization of macrophages	M2 macrophages in islets ↑; blood sugar ↓	continuous expression for several days	I/II	NCT04503278: Used for asthma; No T2DM data available yet	potential hepatotoxicity of LNP delivery may lead to fibrosis	[87,118]
IL-4/albumin nanoparticles	albumin carrier targeted delivery of IL-4 to macrophages induces M2 polarization	Arg1^+^macrophages ↑; pancreatic inflammation ↓; β cells ↑	continuous for several days	preclinical	**-**	may promote fibrosis, and the biological distribution of nanoparticles is uncontrollable	[119]
GLP-1 receptor agonists (liraglutide)	inhibit M1 polarization of macrophages and reduce IL-1 β secretion	insulin sensitivity ↑; β-cell proliferation ↑; weight ↓	inject daily or weekly	approve	LEADER Trial: cardiovascular protection	a risk of gastrointestinal discomfort and pancreatitis	[90]
metformin	activate AMPK and reduce macrophage IL-1 β secretion	CRP, IL-6 ↓; islet macrophage infiltration ↓	oral administration daily	approve	UKPDS Trial: cardiovascular protection	gastrointestinal side effects; Vitamin B12 deficiency	[88,120]

Enhancing the anti-inflammatory function of macrophages helps reestablish islet immune homeostasis through immunomodulatory mechanisms (Table 3). Studies have revealed that extracellular vesicles derived from 3D-cultured human umbilical cord mesenchymal stem cells, which are enriched in miR-146a-5p, promote macrophage polarization toward an M2 anti-inflammatory phenotype by targeting the TRAF6/interleukin-1 receptor-associated kinase 1‌ (IRAK1) signaling axis [84]. In hIAPP transgenic mice, this intervention increased the proportion of M2 macrophages and decreased IL-1β levels in islets. Cell-based studies have demonstrated that vitamin D3 facilitates the polarization of high glucose-induced M1 macrophages toward an M2-like phenotype via the vitamin D receptor (VDR)–peroxisome proliferator-activated receptor γ‌ (PPAR-γ) signaling axis [85]. When exposed to high glucose concentrations in U937 cells, supplementing with vitamin D can better reduce oxidative stress, thereby more effectively inhibiting the release of pro-inflammatory cytokines and chemokines [86]. Novel local delivery approaches, such as mRNA liposomes encoding IL-4/IL-13, significantly upregulate M2 marker genes including Arg1 and found in inflammatory zone 1 (Fizz1) in macrophages [87]. In the streptozotocin (STZ)-induced diabetic mouse model, administration of IL-4 upregulated the expression of M2 macrophage markers in pancreatic islet macrophages and enhanced glucose homeostasis [87].

Modulation of metabolic pathways can indirectly suppress the inflammatory activity of pancreatic islet macrophages (Table 3). Cortés M et al. demonstrated that the antidiabetic agent metformin suppresses chronic inflammation by upregulating the expression of the plasticity factor zinc finger E-box binding homeobox 1‌ (ZEB1) in macrophages [88]. Clinical observations and research have reported a reduction in macrophage infiltration levels in patients with T2DM following long-term metformin treatment [89]. In vivo, glucagon-like peptide-1 receptor (GLP-1R) agonist liraglutide reduced macrophage infiltration and lowered fasting blood glucose in transplanted rats [90]. Fatty acid-binding protein 4 (FABP4), which is predominantly expressed in macrophages and adipose tissue, regulates fatty acid storage and lipolysis, and serves as an important inflammatory mediator [91]. In vitro experiments demonstrate that the FABP4 inhibitor BMS309403 protects pancreatic islet cells from macrophage-mediated cytotoxicity and preserves their insulin secretory capacity [92]. Preclinical data indicate that beta-hydroxybutyrate (BHB) lowers fasting blood glucose levels in rats and modulates macrophage polarization [93].

Targeting pathological crosstalk between macrophages and β-cell offers a promising avenue for precision therapy (Table 3). Clinical trials have demonstrated that the anti-IL-1β monoclonal antibody canakinumab specifically blocks the binding of IL-1β to β-cell IL-1 receptors, effectively lowering fasting glucose levels and improving insulin secretion indices [94]. Canakinumab treatment reduces glycated hemoglobin (HbA1c) in T2DM patients, although infection risks require monitoring [94]. CSF1 is a well-established regulator of macrophage development and differentiation. Experimental results indicate that the CSF-1R inhibitor PLX3397 significantly reduces Pancreatic Islet Macrophage infiltration [95]. However, Hume DA et al. showed that long-term CSF-1R inhibition may cause immunosuppression and other side effects, necessitating careful risk–benefit evaluation [95].

### 1.6 Future research directions and limitations

In recent years, therapeutic strategies directed at macrophages have evolved toward greater precision and sophistication, moving from broad systemic anti-inflammatory approaches to the spatiotemporally controlled "intelligent reprogramming" of pancreatic islet macrophages [18]. Emerging technologies—notably nanotechnology, gene-editing platforms, and advanced biomaterials—offer promising avenues for precise modulation of specific macrophage subpopulations, thereby alleviating islet inflammation and supporting β-cell functional recovery [18]. Nanotechnology-based precision delivery systems occupy a central role in this effort, with ongoing advances reflecting trends toward diversification and intelligent functionality [96].

The fundamental objective of precision nano-delivery systems is the engineering of nanoplatforms that enable efficient targeting and controlled release of therapeutics [97]. Carrier diversity continues to expand: alongside conventional liposomes and polymer nanoparticles, biomimetic nanotechnologies show considerable promise [96]. For instance, nanoparticles coated with macrophage or β-cell membranes exploit homologous targeting mechanisms to improve accumulation within inflamed islets [96]. Engineered exosomes derived from mesenchymal stem cells also serve as natural nanocarriers for anti-inflammatory microRNAs or cytokines, capitalizing on their low immunogenicity and innate homing capability [98,99]. A related frontier involves stimulus-responsive designs, which allow such systems to detect islet microenvironment cues—such as mildly acidic pH or elevated reactive oxygen species—and release payloads in a site-specific manner, thus reducing off-target effects [100,101]. Beyond small molecules and biologics, nanocarriers can also deliver genetic regulators. For example, lipid nanoparticles may carry siRNA to silence pro-inflammatory genes or CRISPR-Cas9 components for in situ gene editing, opening avenues for durable phenotypic reprogramming of macrophages [100]. Furthermore, human islet mimetics based on organoid or organ-on-a-chip platforms facilitate high-throughput screening of macrophage-targeting agents, supporting faster clinical translation [20,102].

Looking forward, therapeutic innovation will extend beyond improved delivery technologies to encompass deeper manipulation of macrophage biology, with immune metabolic intervention emerging as a key strategy. Macrophage functional phenotypes are intimately connected to metabolic pathways: pro-inflammatory M1-like cells predominantly utilize glycolysis, while reparative M2-like phenotypes depend on oxidative phosphorylation and fatty acid oxidation [41]. Consequently, nanoparticle-mediated delivery of metabolic modulators may enable upstream reprogramming of macrophage activity, leading to more sustained anti-inflammatory outcomes [100]. Another promising direction combines cell therapy with synthetic biology—for instance, infusing in vitro-polarized M2-like macrophages or genetically engineered macrophages overexpressing anti-inflammatory factors or growth factors, effectively deploying them as living therapeutics that home to damaged islets [103]. The integration of artificial intelligence with multi-omics data is also poised to accelerate progress. Machine learning algorithms applied to single-cell datasets can identify critical regulators and dynamic shifts in macrophage polarization, informing the selection of personalized therapeutic targets [104].

A major obstacle in the field remains the incomplete understanding of macrophage heterogeneity and functional plasticity, which complicates precise targeting. The conventional M1/M2 classification is increasingly recognized as oversimplified; in vivo, macrophages exist along a functional spectrum, with phenotypes shaped by local signals and subject to dynamic change [105]. In the islets of individuals with T2DM, these cells may display unique transcriptional profiles that deviate from classical activation states [105]. Moreover, pancreatic islet macrophages vary in developmental origin—including embryo-resident populations versus monocyte-derived infiltrates—each with potentially distinct roles [22,35]. Indiscriminate depletion strategies thus risk eliminating beneficial subsets. Macrophage function is also highly context- and time-dependent, meaning the same intervention may produce opposing outcomes at different disease stages [23,24]. At present, reliable biomarkers to stratify patients based on immune microenvironment status are unavailable.

Translation of these strategies from bench to bedside is impeded by several challenges, including species-specific differences, limitations in delivery system performance, and patient heterogeneity. The majority of preclinical studies rely on mouse models, yet significant differences exist between murine and human islets in architecture, immune composition, and disease mechanisms [12,106]. Delivery efficiency remains a critical hurdle: pancreatic islet macrophages are rare, and systemically administered nanocarriers exhibit poor targeting specificity [101]. Long-term biocompatibility of nanomaterials also requires thorough assessment [101]. Furthermore, the substantial variation among T2DM patients in genetics, disease progression, and metabolic features suggests that uniform targeting strategies will likely benefit only specific subgroups [12,106]. Hence, the development of validated biomarkers for patient stratification and individualized therapy design will be crucial for successful clinical adoption.

### 1.7 Discussion

Under physiological and pathological conditions, pancreatic islet macrophages contribute to the maintenance of islet homeostasis as well as to the pathogenesis of T2DM through distinct mechanisms. In the physiological state, embryonic-derived islet-resident macrophages help preserve pancreatic microenvironment homeostasis via immune surveillance, secretion of anti-inflammatory factors such as IL-10 and TGF-β, and metabolic support [24,46]. Under metabolic stress conditions, such as obesity and hyperglycemia, these macrophages can be activated through receptors such as TLR4 and RAGE, triggering the NF-κB and NLRP3 inflammasome signaling pathways and promoting the release of pro-inflammatory cytokines such as IL-1β and TNF-α [33]. These inflammatory mediators directly compromise β-cell function. Specifically, IL-1β downregulates key transcription factors including PDX1 and MafA, thereby suppressing insulin synthesis and secretion [94]. A self-perpetuating pathological cycle emerges between macrophages and β-cell. Here, damaged β-cell release signaling molecules such as IAPP and ATP, which further activate pancreatic islet macrophages and intensify local inflammation [48,71]. Collectively, these mechanisms contribute to the decline in β-cell mass and the failure of insulin secretion, thereby driving the progression of T2DM.

Pancreatic islet macrophages serve not only as important regulators of islet adaptation to systemic metabolic demands, but also as key effector cells in islet dysfunction during T2DM. However, critical scientific questions regarding their phenotypic specificity and spatial heterogeneity remain to be fully answered. Currently, the functional distinction between monocyte-derived macrophages and tissue-islet-resident macrophages is unclear, as are the spatial distribution patterns of different macrophage subsets within the pancreas during disease progression. Beyond established signaling pathways, the potential existence of novel communication mechanisms between macrophages and β-cell also warrants further investigation.

(1) **Pancreatic Islet Macrophages: Guardians of Homeostasis and Drivers of Pathological Transformation**

Under physiological conditions, pancreatic islet macrophages maintain tissue homeostasis and contribute to pancreatic endocrine function. In the healthy pancreas, islet-resident macrophages are derived from embryonic yolk sac hematopoietic progenitors and can self-renew [106]. These cells display considerable plasticity in response to local microenvironmental cues and contribute to tissue homeostasis by clearing apoptotic debris and secreting anti-inflammatory factors such as IL-10 and TGF-β [106]. They also support β-cell proliferation and islet vascularization through the release of VEGF and PDGF [27]. In the context of T2DM, chronic hyperglycemia and lipotoxicity drive monocyte-derived macrophages toward a pro-inflammatory M1 phenotype [58]. Concurrently, the expression of M2 markers is downregulated, while pro-inflammatory cytokines such as IL-1β and TNF-α are abundantly secreted. These changes inhibit the key β-cell transcription factor PDX1, induce ER stress, and ultimately impair insulin synthesis and secretion [107]. Given these dynamics, targeted modulation of functionally distinct macrophage subsets has emerged as a promising therapeutic strategy for T2DM [107].

(2) **Heterogeneity of Islet Macrophage Distribution: Spatial Localization and Functional Specialization**

ScRNA-seq have revealed substantial functional heterogeneity among pancreatic islet macrophages in T2DM, extending beyond the conventional M1/M2 classification framework [12]. Recent analyses indicate the presence of at least three functionally distinct macrophage subsets in the pancreas. The first is a pro-inflammatory CD9^+^ population that highly expresses NLRP3 and IL-1β, forming an “inflammatory cuff” around the islet [7]. The second is a CD11c^+^ MHC-II^+^ subset situated at the vascular interface and specialized in antigen presentation [36]. The third includes TREM2^+^ LYVE1^+^ lipid-associated macrophages that activate the HIF-1α/IL-1β axis via succinate metabolism [108]. Notably, Baer JM et al. demonstrated that lipid-related subsets such as TREM2^+^ macrophages integrate inflammatory signaling with metabolic reprogramming, representing a novel activation state independent of classical polarization paradigms [108]. This refined cellular heterogeneity map provides a critical molecular foundation for developing precise targeted therapeutic strategies.

(3) **The Macrophage-β-Cell Communication Network: Known and Unknown Mechanisms**

Bidirectional communication between pancreatic islet macrophages and β-cell is mediated through diverse molecular mechanisms, including cytokine signaling, metabolic exchange, and direct cell–cell contact. Well-characterized communication pathways involve specific cytokine axes. For instance, IL-1β activates NF-κB signaling via the IL-1R/MyD88-dependent pathway, leading to the suppression of insulin gene transcription [13]. Direct cellular interactions also contribute. For example, integrin β2 binding to intercellular adhesion molecule-1‌ (ICAM-1) can trigger calcium-mediated regulation of insulin secretion [38]. Metabolite exchange represents another layer of interaction: lactate imported via monocarboxylate transporter 1 (MCT1) suppresses oxidative phosphorylation in β-cell [109]. Together, these interactions constitute a sophisticated network of intercellular crosstalk.

The underlying mechanisms, which remain to be fully elucidated, may involve exosome-mediated transfer of microRNAs such as macrophage-secreted miR-155 contributes to impairments in β-cell GSIS and insulin biosynthesis by targeting PDX1 [110]. Long non-coding RNA (lncRNA) represent an emerging frontier in genomic regulation research. Recent evidence indicates that lncRNA participate in the modulation of inflammatory responses through multiple mechanisms, including epigenetic, transcriptional, and post-transcriptional levels of regulation [111]. Additionally, neuroimmune interactions. For example, the modulation of inflammatory polarization by norepinephrine via β2-adrenergic receptors (β2-AR) on macrophages—represent potential novel regulatory pathways [7]. A thorough dissection of these mechanisms will require further investigation using spatial multi-omics technologies and advanced in vivo imaging approaches.

### 1.8 Conclusion

Pancreatic islet macrophages play a dichotomous role in the pathogenesis of T2DM. In addition to contributing to islet inflammatory responses, they are also essential for maintaining islet homeostasis. A disturbance in this functional equilibrium exacerbates β-cell failure through immune metabolic crosstalk. Conversely, modulating macrophage polarization and remodeling the local microenvironment offer promising therapeutic options. Advances in research technologies, particularly the integration of single-cell analysis, nanomedicine, and artificial intelligence, are expected to overcome current limitations in understanding spatiotemporal cellular heterogeneity. Such progress will likely accelerate the translation of mechanistic insights into innovative clinical treatments.

## CRediT authorship contribution statement

**Zhongpeng Qiu:** Writing – original draft. **Dejing Shang:** Writing – review & editing.

## Conflict of interest

The authors declare that there are no conflicts of interest.

## Figures and Tables

**Table:** 

List of Abbreviations			
Abbreviation	Full Form	Abbreviation	Full Form
T2DM	type 2 diabetes mellitus	iNOS	inducible nitric oxide synthase
PDGF	platelet-derived growth factor	ROS	reactive oxygen species
VEGF	vascular endothelial growth factor	PDX1	pancreatic duodenal homeobox factor-1
IL-1β	interleukin-1β	ATP	adenosine triphosphate
TNF-α	tumor necrosis factor α	DAMPs	damage-associated molecular patterns
TLR4	Toll-like receptor 4	PI3K	Phosphatidylinositol 3-kinase
NLRP3	NOD-like receptor family pyrin domain-containing 3	Akt	phosphatidylinositol-dependent kinase B
CCL2	monocyte chemotactic protein-1	FoxO1	Fork head box O1
CCR2	chemokine receptor 2	Bcl-2	B-cell lymphoma/leukemia-2
CSF-1	colony-stimulating factor 1	EGF	epidermal growth factor
CSF-1R	colony-stimulating factor 1 receptor	ERK	Extracellular Regulated Kinases
scRNA-seq	Single cell RNA sequencin	MyD88	myeloid differentiation factor 88
IGF	insulin-like growth factor	IL-6	interleukin-6
M-CSF	macrophage colony-stimulating factor	TNTs	tunneling nanotubes
Arg1	arginase-1	GSIS	glucose-stimulated insulin secretion
CD206	Scavenger receptor 206	HIF-1α	Hypoxia-inducible factor 1-alpha
IL-10	interleukin-10	MafA	Musculoaponeurotic fibrosarcoma oncogene homolog A
FFA	free fatty acids	ER	Endoplasmic reticulum
CD11c	scavenger receptor 11c	JNK	c-Jun N-terminal kinase
CD68	scavenger receptor 68	MFN2	mitochondrial fusion protein 2
db/db	leptin receptor deficiency	COX-2	cyclooxygenase-2
HFD	high-fat diet	PGE_2_	prostaglandin E_2_
AGE	advanced glycation end product	EP3R	prostaglandin E_2_ receptor
TLR	Toll-like receptor	hIAPP	human islet amyloid polypeptide
NF-κB	nuclear factor kappa B	NO	nitric oxide
MAPK	mitogen-activated protein kinase	IAPP	islet amyloid polypeptide
TGF-β	transforming growth factor β	IRE1α	inositol-requiring enzyme 1α
IL-4	interleukin-4	XBP1	X-box binding protein 1
IL-13	interleukin-13	BiP	binding immunoglobulin protein
LPS	lipopolysaccharides	AP-1	activator protein 1
DIO	diet induced obesity	RAGE	receptor for advanced glycation end products
GLUT1	glucose transporter type 1	PDK4	pyruvate dehydrogenase kinase 4
PKC	protein kinase C	GPR132	G protein-coupled receptor 132
STAT1	signal transducer and activator of transcription 1	IRAK1	interleukin-1 receptor-associated kinase 1
IRF5	interferon regulatory factor 5	VDR	vitamin D receptor
IRF8	interferon regulatory factor 8	PPAR-γ	peroxisome proliferator-activated receptor γ
PA	Palmitate	Fizz1	found in inflammatory zone 1
TIR	Toll/interleukin-1 receptor	STZ	streptozotocin
IRAK	IL-1 receptor-associated kinase	ZEB1	zinc finger E-box binding homeobox 1
TRAF6	TNF receptor-associated factor 6	GLP-1R	glucagon-like peptide-1 receptor
TAK1	transforming growth factor-β-activated kinase 1	GK	Goto-Kakizaki
CD36	scavenger receptor 36	FABP4	fatty acid-binding protein 4
mtDNA	mitochondrial DNA	BHB	β-hydroxybutyrate
cGAS	cyclic GMP-AMP synthase	HbA1c	glycated hemoglobin
STING	stimulator of interferon genes	ICAM-1	intercellular adhesion molecule-1
UPR	unfolded protein response	MCT1	monocarboxylate transporter 1
PERK	protein kinase R-like endoplasmic reticulum kinase	SIRT2	sirtuin 2
ATF6	activating transcription factor 6	GSK-3β	glycogen synthase kinase-3β
TXNIP	thioredoxin-interacting protein	lncRNA	long non-coding RNA
CHOP	C/EBP homologous protein	β2-AR	β2-adrenergic receptors
TRX	thioredoxin	GPCR	G Protein-Coupled Receptor
mtROS	mitochondrial reactive oxygen species	HOMA-IR	Homeostatic Model Assessment of Insulin Resistance
ox-LDL	Oxidized low-density lipoprotein	CRP	C-reactive protein
LOX-1	lectin-like oxidized low-density lipoprotein receptor 1		
VDR	vitamin D receptor		
